# Bibliometric analysis of genetically engineered bacteria in tumor therapy

**DOI:** 10.3389/fmicb.2026.1794032

**Published:** 2026-04-21

**Authors:** Yanmei Zhu, Yilin Yin, Yaqi Li, Qinghua Wang, Yu Chen, Ke Gu, Rui Yang, Min Zhou, Daozhen Chen

**Affiliations:** 1 Wuxi Maternity and Child Health Care Hospital, Jiangnan University, Wuxi, Jiangsu, China; 2 Wuxi School of Medicine, Jiangnan University, Wuxi, Jiangsu, China; 3 Department of Radiotherapy, Affiliated Hospital of Jiangnan University, Wuxi, Jiangsu, China; 4 Wuxi Higher Health Vocational Technology School, Wuxi, Jiangsu, China; 5 Department of Breast Surgery, Wuxi Maternity and Child Health Care Hospital, Jiangnan University, Wuxi, Jiangsu, China

**Keywords:** bacterial therapy, bibliometrics, gene editing, genetically engineered bacteria, tumor

## Abstract

The utilization of bacteria for tumor treatment has a long-standing history. In recent years, with the continuous advancement of genetic engineering technologies, genetically engineered bacteria have attracted widespread attention in tumor-related research; however, to date, no bibliometric analysis has been reported for this field. In this study, bibliometric methods were employed to comprehensively evaluate the current research status, hotspots, and development trends of genetically engineered bacteria in the tumor field. Studies focusing on genetically engineered bacteria and tumors were retrieved from the Web of Science Core Collection (WOSCC) database and the Scopus database, and bibliometric and visualization analyses were conducted using R, VOSviewer, Scimago Graphica, and CiteSpace. Finally, we discuss the current limitations and challenges associated with the application of genetically engineered bacteria in the tumor field. Future research should focus on improving the safety, targeting capability, and clinical feasibility of engineered bacteria, as well as systematically investigating the interaction mechanisms among different therapeutic modalities, in order to promote the clinical translation of genetically engineered bacteria for tumor treatment.

## Introduction

Cancer remains one of the leading causes of death worldwide, characterized by aberrant proliferation, invasive metastasis, and persistent destruction of normal tissues. According to the World Health Organization, there were approximately 20 million new cancer cases and 9.7 million cancer-related deaths globally in 2022, and this number is projected to rise to 35 million new cases by 2050 (Bray et al., 2024). Although conventional therapeutic modalities—including surgery, radiotherapy, and chemotherapy—have improved patient survival to some extent, their efficacy is often limited by tumor heterogeneity, drug resistance, insufficient targeting, and systemic toxicities (Rui et al., 2023; Yu et al., 2019). Consequently, the development of more precise, effective, and less toxic therapeutic strategies remains a central scientific challenge and an urgent clinical need in oncology.

In recent years, emerging approaches such as immunomodulation, molecular targeting, and gene therapy have been introduced and have demonstrated potential clinical value in selected tumor types (Wang et al., 2019; Gao et al., 2021). Nevertheless, these therapies still encounter multiple bottlenecks in real-world clinical application, including adaptive and tolerant mechanisms of tumor cells, immune evasion and immunosuppressive microenvironments, significant inter-patient variability in efficacy, and high treatment costs, resulting in a considerable proportion of patients failing to achieve stable and sustainable clinical benefit. Given these limitations, the development of novel therapeutic systems has become crucial for advancing oncological treatments. These systems aim to achieve high-selectivity enrichment, controlled release, and multi-mechanism synergy *in vivo*. Bacteria-mediated tumor therapy offers unique advantages and has gained rapid momentum with the development of genetic engineering and synthetic biology tools (Ma et al., 2017). Tumor tissues often exhibit abnormal vasculature, local hypoxia, and necrotic areas. These conditions create a favorable environment for bacterial colonization, leading to a “tumor-tropic” enrichment effect. Researchers have rationally engineered bacteria through gene editing and synthetic biology design to enable targeted colonization within the tumor microenvironment and to execute therapeutic functions: on the one hand, they can express or deliver cytotoxic/oncolytic payloads, enzymatic products, or small-molecule drugs to achieve direct tumor cell killing; on the other hand, they can present antigens, secrete cytokines, and activate T-cell-mediated immune effects, thereby reshaping the tumor immune microenvironment. In addition, engineered bacteria can serve as gene-tool delivery platforms, carrying CRISPR editing elements or siRNA nucleic acids to trigger intratumoral gene-regulatory circuits and therapeutic responses (Song et al., 2024; Chen et al., 2021; Nguyen et al., 2023).

**Figure 1:**
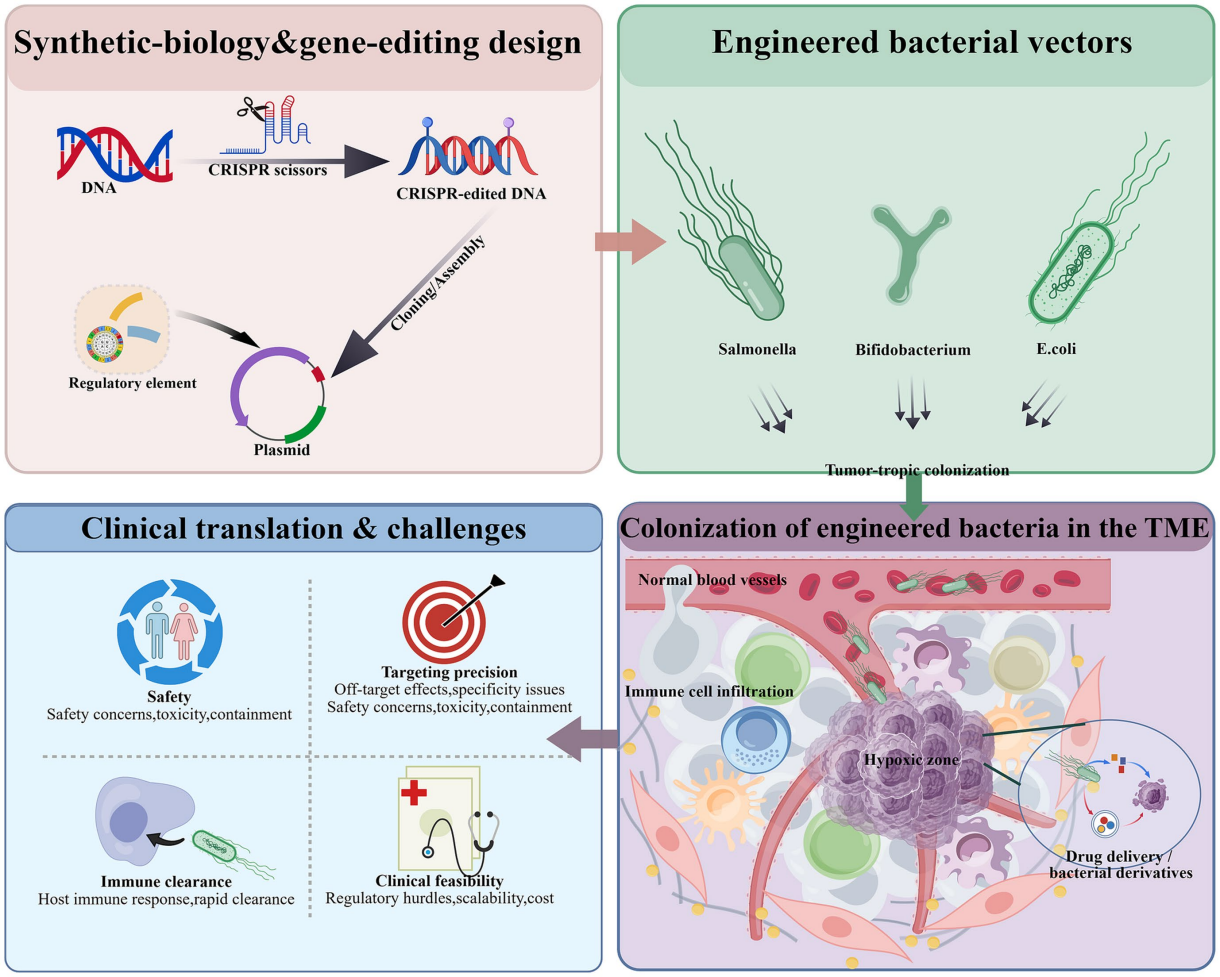
Conceptual framework of synthetic-biology-driven engineered bacterial strategies for cancer therapy [created with BioGDP.com (Jiang et al., 2025)].

To visualize the technical routes and therapeutic outputs of this field more intuitively, we introduce a conceptual framework diagram (Figure 1). As illustrated, engineered bacterial tumor therapy generally follows a chained process: “synthetic biology/gene-editing design → engineered bacterial vector construction (e.g. *E. coli*, Bifidobacterium, or Salmonella) → tumor-tropic colonization → intratumoral release of drugs/bacterial derivatives”. This framework not only summarizes the overall evolution from “bacterial modification per se” to “programmable delivery systems”, but also underscores the critical influence of the tumor microenvironment—such as hypoxic zones and immune-cell infiltration—on the functional performance and therapeutic variability of engineered bacteria.

Despite remarkable progress in bacteria-mediated tumor therapy, its clinical translation is constrained by multiple factors, including inadequate in-vivo fitness and competitive capacity, limited targeting accuracy and off-target risks, host immune clearance, potential toxicity and biosafety control, as well as challenges in scalable manufacturing and regulatory compliance (Lin et al., 2024). Against this backdrop, systematically mapping the research architecture, hotspot themes, and developmental trends of this field is essential for identifying key technological bottlenecks, grasping frontier directions, and accelerating clinical translation. Bibliometrics enables quantitative visualization of research outputs, collaboration networks, knowledge bases, and hotspot evolution, providing relatively objective evidence for disciplinary development trajectories and future trends (Mayr and Scharnhorst, 2015). However, to date, systematic bibliometric studies specifically focusing on “genetically engineered bacteria in tumor therapy” remain scarce. Here, by performing bibliometric and visual analyses of relevant publications indexed in the Web of Science Core Collection (WOSCC) and Scopus databases, we provide a comprehensive evaluation of this emerging domain. Our aims are to reveal the overall developmental landscape, identify core countries, key authors, and high-impact journals, pinpoint research hotspots and frontier topics, and—by integrating current limitations and challenges—propose future research directions, thereby offering an evidence-based reference for subsequent investigations and clinical translation of engineered bacterial cancer therapy. [In this study, the use and declaration of artificial intelligence adhere to relevant norms (Riaz et al., 2025)].

## Methods

### Data acquisition and search strategy

To ensure comprehensive coverage of research on genetically engineered bacteria in tumor therapy, a dual-database retrieval strategy was adopted using the Web of Science Core Collection (WoSCC) and Scopus. The literature search was conducted on December 12, 2025, and the retrieved records represent the database status on that date. Using both databases helps reduce potential disciplinary bias and improves the completeness of the bibliometric dataset.

**Figure 2:**
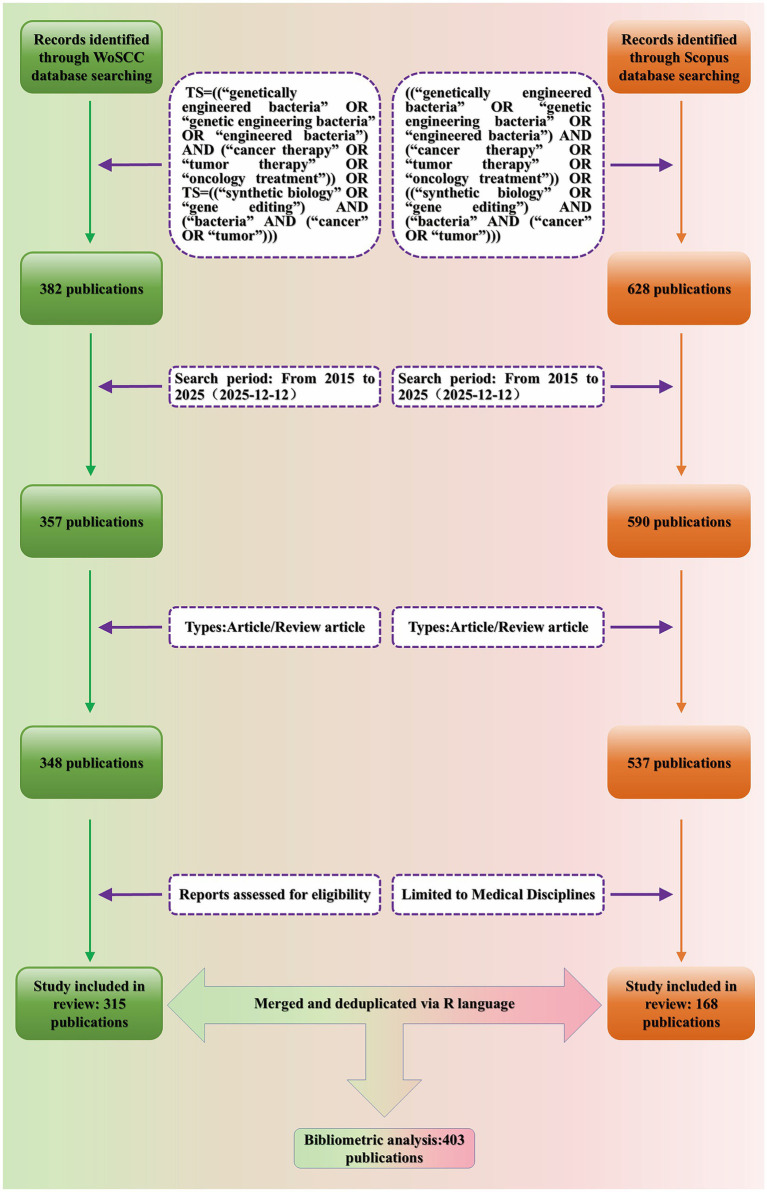
Flowchart of the literature screening process.

The search strategy was constructed around three conceptual components: genetically engineered bacteria as the research object, cancer therapy as the application context, and enabling technologies such as synthetic biology and gene editing. The search was performed in the Title, Abstract, and Keywords fields, and the following query was applied: [(“genetically engineered bacteria” OR “genetic engineering bacteria” OR “engineered bacteria”) AND (“cancer therapy” OR “tumor therapy” OR “oncology treatment”)] OR [(“synthetic biology” OR “gene editing”) AND (“bacteria” AND (“cancer” OR “tumor”))]. Only Article and Review publications were included, and the time span was restricted to 2015–2025 to capture recent developments in this field (Figure 2). The detailed search strategy is provided in Supplementary Table S1.

### Data processing and bibliometric analysis

WoSCC records were exported in plain text format with full records and cited references, whereas Scopus records were exported in CSV format. The datasets were subsequently imported into the R environment for data cleaning and integration.

Data preprocessing was primarily conducted using the bibliometrix package together with functions from the base R environment. Duplicate records between the two databases were identified and merged using the mergeDbSources() function based primarily on unique identifiers such as DOI, followed by additional verification of key fields (e.g., titles) using the duplicated() function to minimize mismatches caused by formatting differences. To address variations in author name formats across databases, author names were further standardized using custom R scripts. Surnames were preserved, and given names were normalized to full-name forms whenever possible. When full names could not be reliably inferred, the original initials were retained but consistently grouped under the same author identity. After automated processing, high-frequency author records were manually checked to correct rare unresolved variants, ensuring the reliability of author-level bibliometric analyses. The cleaned and deduplicated dataset covering publications from 2015 to 2025 served as the basis for subsequent bibliometric analyses and visualization.

Bibliometric analyses and network visualizations were conducted using VOSviewer, CiteSpace, and Scimago Graphica. Unless otherwise specified, default parameter settings of the software were applied. Threshold values were set to ensure clarity and interpretability of the networks; for example, countries with at least three publications were included in the country collaboration analysis, and keyword co-occurrence thresholds were adjusted according to dataset size. Labels in the visualizations generated by VOSviewer and CiteSpace follow the original database records.

## Results

### Analysis of general trend

**Figure 3:**
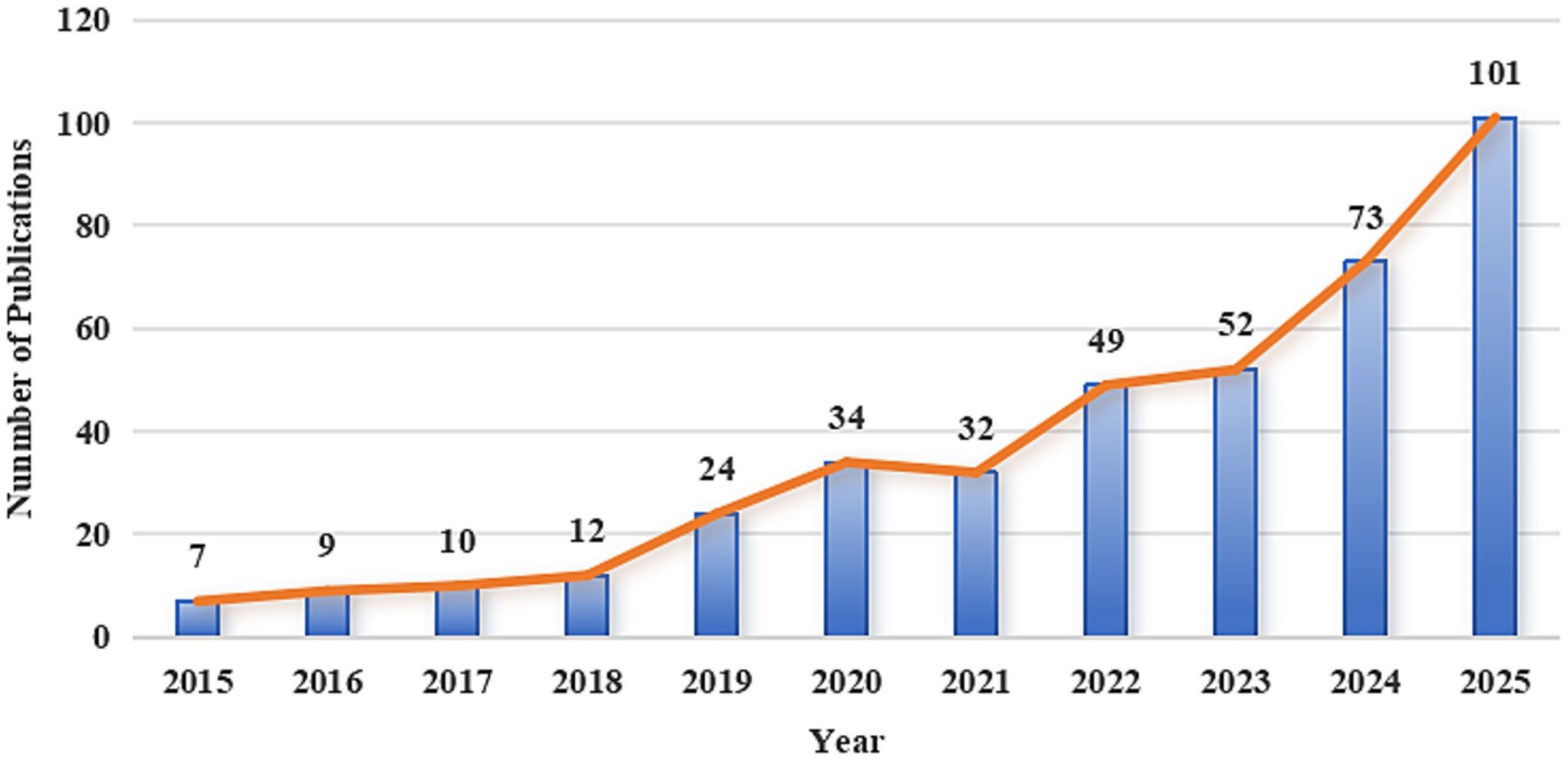
Annual publication trends in genetically engineered bacteria for tumor therapy from 2015 to 2025.

The 403 papers employed in this study originated from 2,269 authors affiliated with 963 institutions across 48 countries, were published in 232 journals, and cited 23,569 references drawn from 4,326 journals. Between 2015 and 2025, the annual scientific output exhibited an overall upward trajectory, indicating heightened attention toward genetically engineered bacteria in the field of oncology (Figure 3).

### Analysis of countries/regions

**Figure 4:**
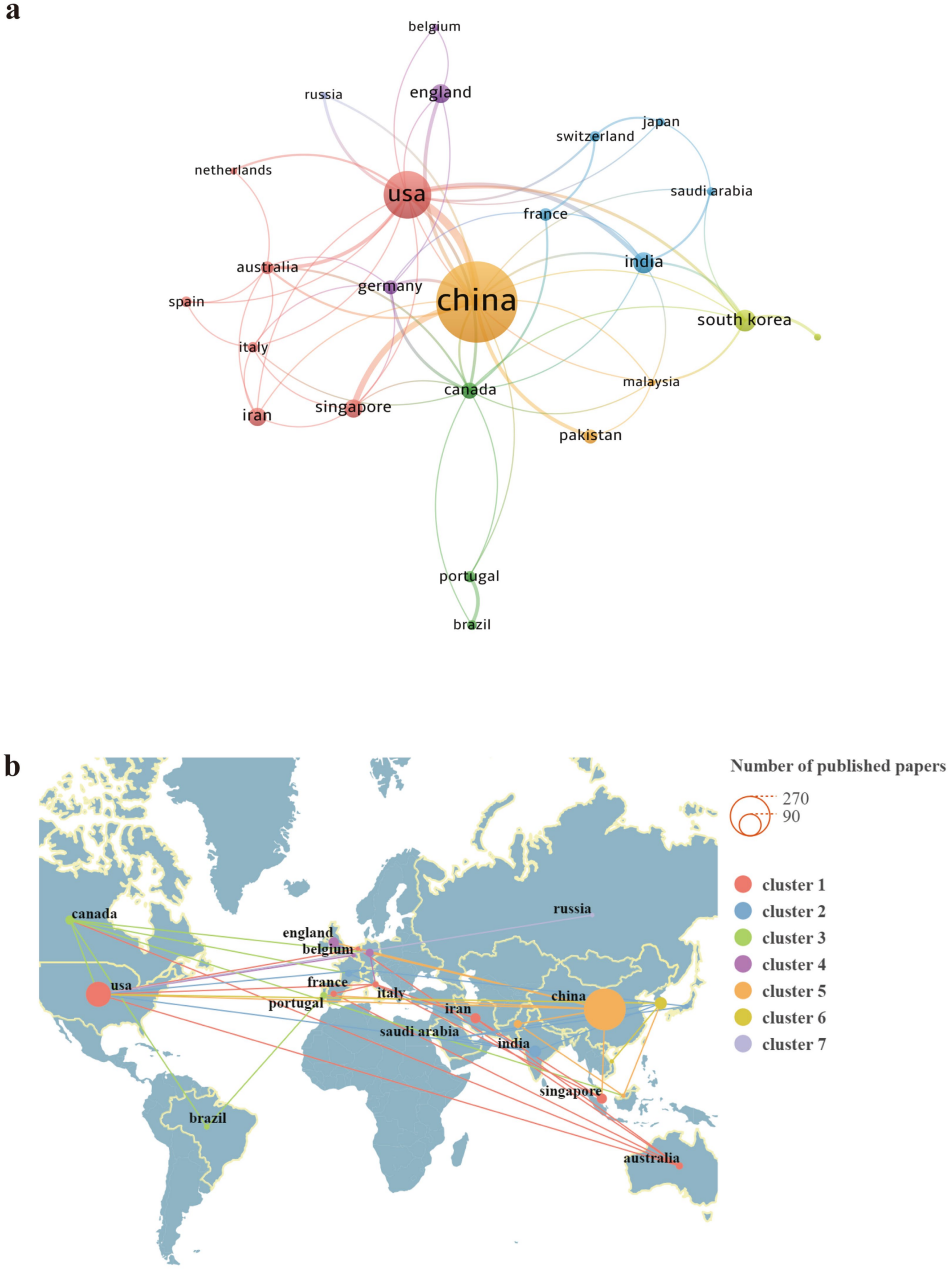
Country distribution of publications in genetically engineered bacteria for tumor therapy. **(a)** Country collaboration network; node size represents publication output and node colors indicate collaboration clusters. **(b)** Global distribution of publications. Labels in the visualizations generated by VOSviewer and CiteSpace follow the original database records.

This study analyzed 48 countries with the aim of identifying those with prominent contributions to research on the application of genetically engineered bacteria in tumors. Countries with three or more publications were visualized using VOSviewer and Scimago Graphica (Figure 4). A quantitative summary of the main bibliometric indicators of the most productive countries, including publications, total citations, average citations per article, and total link strength, is presented in Table 1. In Figure 4a, larger circular nodes indicate higher publication output; links between nodes represent the strength of associations, with thicker links denoting a greater number of co-authored publications between two countries; node colors correspond to different collaboration clusters identified by the clustering algorithm, representing groups of countries with relatively stronger collaborative relationships. Figure 4b is based on a world map and distinguishes collaboration clusters using links of different colors, providing a more intuitive representation of the global spatial distribution of academic collaborations among countries. As shown by the visualization in Figure 4, publications on genetically engineered bacteria in the tumor field are mainly concentrated in East Asia, North America, and Europe. Among these regions, East Asia accounts for a dominant share of publications, significantly exceeding that of North America, primarily driven by the high output from China. At the national level, China and the United States are the leading contributors in this field, demonstrating clear advantages in knowledge production. International collaboration visualization indicates that China and the United States maintain the closest cooperative relationship, while also exhibiting strong collaborative ties with multiple European countries; collaborations among other countries are relatively dispersed. Within Europe, countries have formed relatively dense regional collaboration networks, whereas countries such as Australia and Canada participate in research across multiple collaboration clusters. These findings indicate that research on the application of genetically engineered bacteria in tumors has formed an international scientific landscape centered on China and the United States, characterized by the coexistence of regional coordination and cross-regional collaboration.

**Table 1:** Top 10 most productive countries in genetically engineered bacteria-based tumor therapy.

Rank	Country	Documents	Citations	Avg citations	Total link strength
1	China	268	5,304	19.79	44
2	USA	97	7,380	76.08	42
3	South Korea	23	691	30.04	13
4	India	22	305	13.86	14
5	England	17	1,149	67.59	7
6	Iran	16	469	29.31	5
7	Singapore	16	872	54.50	13
8	Canada	13	1,081	83.15	19
9	Germany	10	657	65.70	11
10	Pakistan	10	232	23.20	5

### Analysis of authors

**Figure 5:**
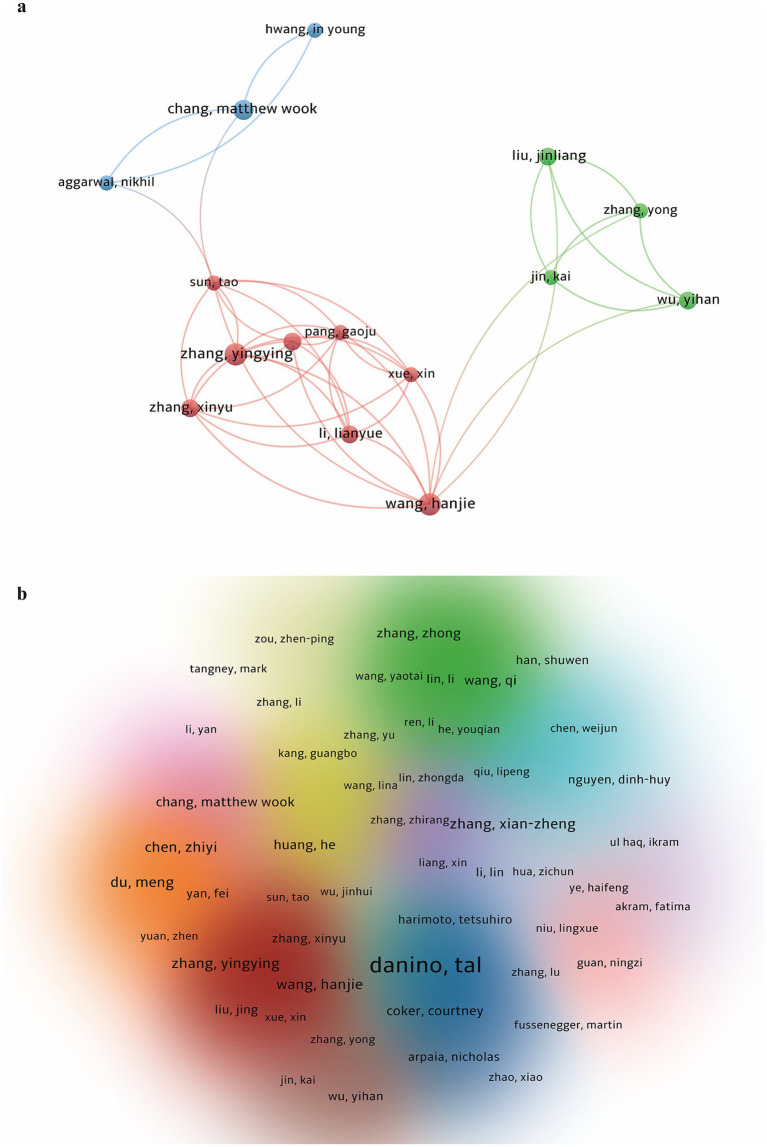
Author collaboration networks in genetically engineered bacteria for tumor therapy. **(a)** Focused view of 15 core authors, mainly from the Asia–Pacific region. Node size indicates publication frequency and node colors represent collaboration clusters. **(b)** Overview of the full network of 74 authors, showing global and regional collaborations.

Lotka’s law describes the distribution pattern of the number of papers published by scientific researchers, indicating that the number of authors publishing X papers is inversely proportional to the square of X (Lotka, 1926). The renowned scholar Price further proposed in his work that the number of highly productive authors is approximately equal to the square root of the total number of authors. These laws are widely used in bibliometrics to identify core authors, thereby revealing representative scholars and the central research forces within a given field. Table 2 presents the top five most productive authors in this research area. Among them, Danino T ranks first, having published a total of 14 papers between 2015 and 2025, which collectively received 2,303 citations, with an average of 164.5 citations per paper. Zhang XZ ranks second, with six publications and 195 total citations, corresponding to an average of approximately 32 citations per paper. These results indicate that both scholars hold relatively authoritative positions in the field of genetically engineered bacteria and cancer research. According to the threshold formula for core authors proposed by Price (m = 0.749 × $\sqrt{n_{\max}}$ , where m represents the minimum number of publications required to be considered a core author), the minimum publication threshold for core authors in this field is m = 0.749 × $\sqrt{n_{\max}}$ ≈ 2.80, given that $\;n_{\max}$ =14 as determined by VOSviewer. Therefore, authors with three or more publications (including three) were identified as core authors in this field. Based on VOSviewer statistics, a total of 74 core authors were identified, collectively contributing 275 publications, accounting for 68.2% of the total output, which exceeds the 50% criterion proposed by Price. To visually illustrate the collaborative relationships among these core authors, we constructed two complementary author collaboration networks using VOSviewer (Figure 5). Figure 5a presents a focused view of the most tightly connected subnetwork, highlighting the strongest collaborative relationships among 15 authors. This subnetwork reveals a closely knit research group primarily concentrated in the Asia–Pacific region, including key scholars such as Chang MW (National University of Singapore), Hwang IY, and Zhang Y. The dense connections within this group indicate highly active regional collaboration. Figure 5b provides a comprehensive overview of the entire core author network, encompassing all 74 core authors. The density visualization indicates that the field has developed into a globally distributed research landscape characterized by strong regional collaborations, further confirming that research on genetically engineered bacteria in oncology has formed a stable and diverse author collaboration network.

**Table 2:** Top 10 authors in genetically engineered bacteria-based tumor therapy.

Rank	Author	Documents	Citations	Average citation/publication
1	Danino T	14	2,303	164.50
2	Zhang XZ	6	195	32.50
3	Chen ZY	6	186	31.00
4	Du M	6	186	31.00
5	Wang HJ	6	152	25.33

### Analysis of journals

**Figure 6:**
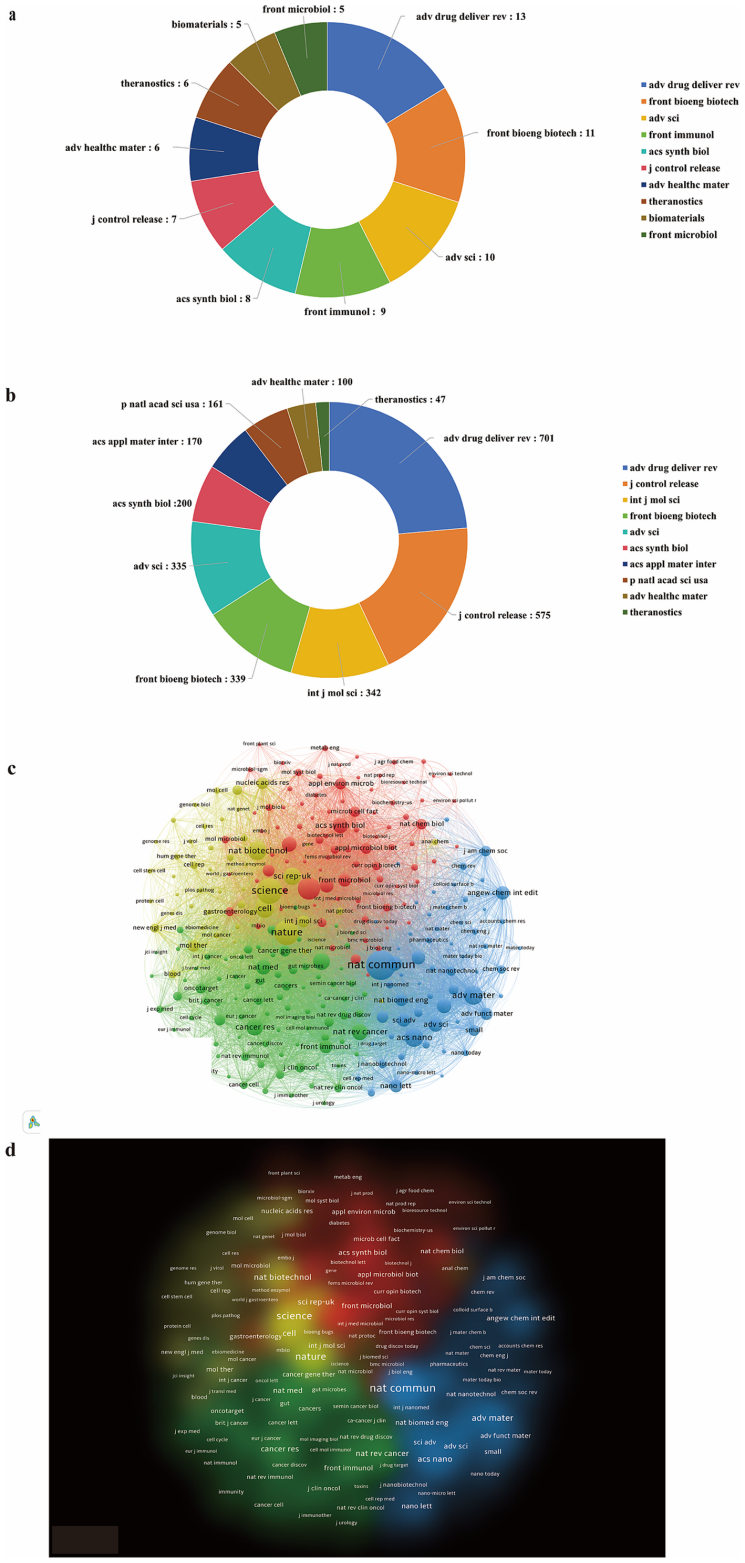
Journals related to genetically engineered bacteria for tumor therapy. **(a)** Top 10 publishing journals. **(b)** Top 10 cited journals. **(c)** Journal co-citation network. **(d)** Journal density map.

Since 2015, a total of 232 journals have published papers related to the application of genetically engineered bacteria in tumor therapy. Table 3 summarizes key information for the top 20 journals by publication output, all of which are classified as JCR Q1 journals, further confirming the vigorous development of this research direction in recent years. As shown in Figure 6a, the top 10 journals in terms of publication output in the field of genetically engineered bacteria–based tumor therapy published a total of 80 papers between 2015 and 2025. Among them, Advanced Drug Delivery Reviews ranked first with 13 publications, followed by Frontiers in Bioengineering and Biotechnology with 11 publications, Advanced Science with 10 publications, and Frontiers in Immunology with 9 publications. However, the ranking by citation frequency does not fully coincide with that by publication output (Figure 6b). The most influential journal, Advanced Drug Delivery Reviews, received more than 701 citations. Journal of Controlled Release ranked second in total citations with 575 citations despite publishing only seven papers, followed by International Journal of Molecular Sciences (342 citations), Frontiers in Bioengineering and Biotechnology (339 citations), and Advanced Science (335 citations). These results indicate that Advanced Drug Delivery Reviews, Frontiers in Bioengineering and Biotechnology, and Journal of Controlled Release represent the most influential publication platforms in the field of genetically engineered bacteria for tumor therapy.

**Table 3:** Top 20 journals (by paper count).

Rank	Journal	Citations	Country	IF	JCR
1	Adv. Drug Deliv. Rev.	701	Netherlands	17.6	Q1
2	Front. Bioeng. Biotechnol.	339	England	4.8	Q1
3	Adv. Sci.	335	USA	14.1	Q1
4	Front. Immunol.	130	USA	5.9	Q1
5	ACS Synth. Biol.	200	USA	3.9	Q1
6	J. Control. Release	575	Netherlands	11.5	Q1
7	ACS Appl. Mater. Interfaces	170	USA	8.2	Q1
8	Adv. Healthc. Mater.	100	Germany	9.6	Q1
9	Proc. Natl. Acad. Sci. U.S.A.	161	USA	9.1	Q1
10	Theranostics	47	Australia	13.3	Q1
11	ACS Nano	166	USA	16.1	Q1
12	Biomaterials	90	Netherlands	12.9	Q1
13	Curr. Opin. Biotechnol.	249	England	7	Q1
14	Front. Microbiol.	151	Switzerland	4.5	Q1
15	Mater. Today Bio	56	England	10.2	Q1
16	Acta Biomater.	155	England	9.6	Q1
17	Angew. Chem. Int. Ed.	55	Germany	17	Q1
18	Cell Rep. Med.	57	USA	10.6	Q1
19	J. Nanobiotechnol.	42	England	12.6	Q1
20	Nat. Commun.	684	England	15.7	Q1

Subsequently, we conducted an in-depth analysis of 284 related journals with more than 30 citations each (Figures 6c,d). The results revealed that these journals form four distinct academic clusters. In the visualization maps, node size is proportional to publication volume, while connecting lines represent the strength of co-citation relationships between journals. Journals focusing on the application of genetically engineered bacteria in tumor research exhibit pronounced co-citation patterns, indicating strong intellectual linkages and thematic coherence within this research domain.

### Analysis of keywords

As a core element of academic publications, keywords concisely reflect the central content and disciplinary orientation of a study. Analysis of keyword co-occurrence relationships enables effective identification of current research hotspots. According to the statistics presented in Table 4, the top ten most frequently occurring keywords are, in descending order: synthetic biology, bacteria, cancer, bacterial derivatives, genetically-engineered bacteria, design principles, diverse applications, gene editing, physiochemically-engineered bacteria, and human. These high-frequency terms collectively delineate the major research trajectories of genetically engineered bacteria in tumor therapy.

**Table 4:** Top 10 keywords of genetically engineered bacteria in the field of tumor research.

Rank	Keyword	Occurrences	Total link strength
1	Synthetic biology	121	149
2	Bacteria	90	149
3	Cancer	82	143
4	Bacterial derivatives	81	370
5	Genetically-engineered bacteria	79	368
6	Design principles	76	366
7	Diverse applications	76	366
8	Gene editing	76	118
9	Physiochemically-engineered bacteria	76	366
10	Human	75	169

**Figure 7:**
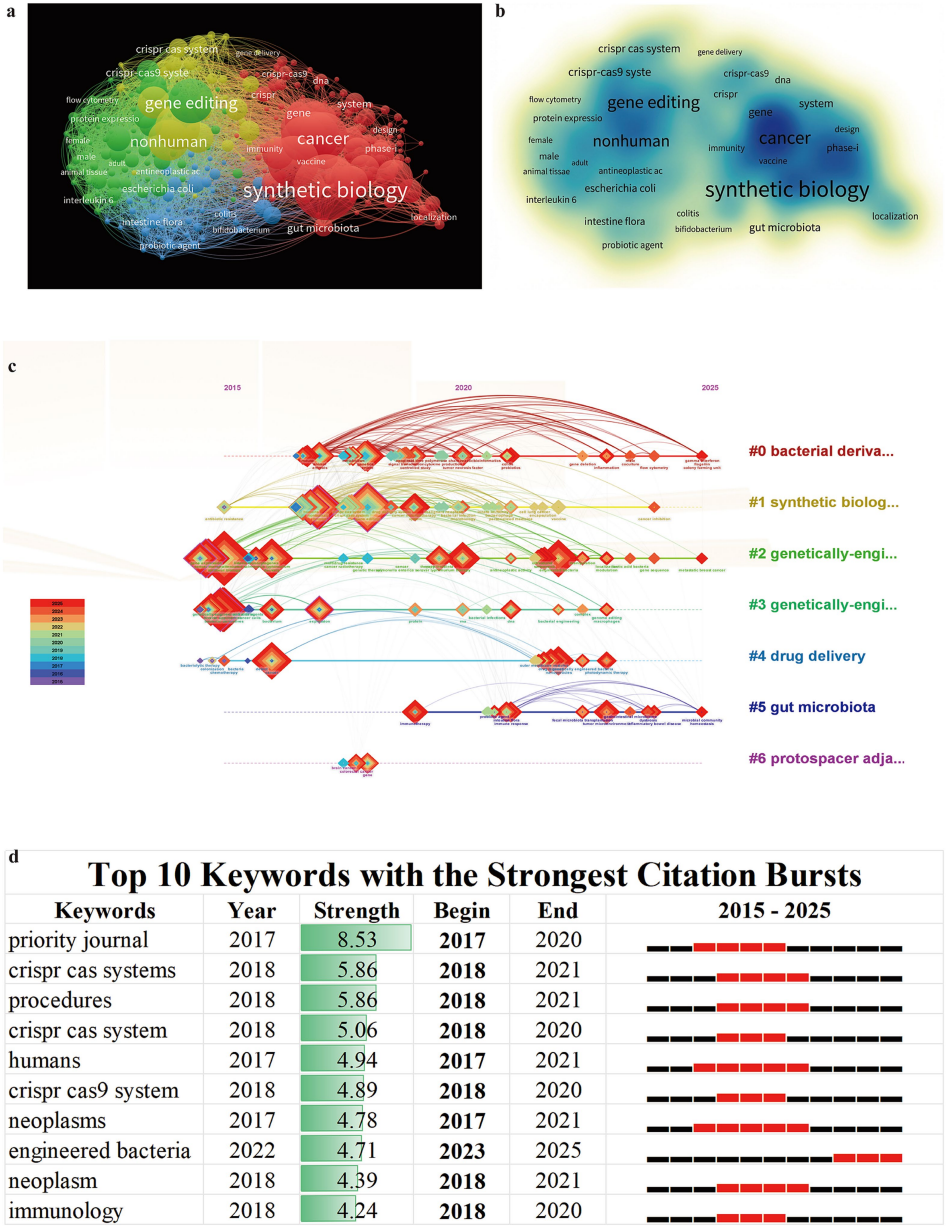
Keyword analysis in genetically engineered bacteria for tumor therapy. **(a)** Keyword co-occurrence network. **(b)** Keyword density map. **(c)** Timeline view of author keyword evolution from 2015 to 2025. **(d)** Top 10 keywords with the strongest citation bursts; black bars indicate keyword occurrence and red bars indicate burst periods.

Using the bibliometric tool VOSviewer, we analyzed the topological structure of the keyword network and visualized the results. Based on a predefined threshold (occurrence ≥5), 279 high-frequency keywords were screened from an initial set of 3,644 terms to construct the co-occurrence network. As shown in Figure 7a, the keyword co-occurrence network consists of four major clusters, each represented by a distinct color. Keywords in the red and green clusters are primarily associated with genetic modification technologies (e.g., synthetic biology, gene editing, CRISPR-Cas9 system). In addition, keywords in the red cluster jointly point to disease models and therapeutic applications together with those in the yellow cluster (e.g., cancer, immunity, vaccinia, phase I, nonhuman). Keywords in the blue and green clusters (e.g., *E. coli*, Bifidobacterium, probiotic agent) are mainly related to engineered bacterial vectors. In the density visualization map (Figure 7b), high-intensity signals for synthetic biology, cancer, gene editing, and nonhuman directly highlight current research hotspots. Overall, the field of genetically engineered bacteria for tumor therapy has formed a research paradigm strongly driven by synthetic biology, with CRISPR-based gene editing as the primary technological approach, highly focused on cancer treatment and predominantly remaining at the nonhuman stage (Jeong et al., 2013; Zheng et al., 2023; Hao et al., 2024).

The timeline of the keyword co-citation network, generated using CiteSpace, illustrates the historical evolution of research hotspots and thematic shifts. As shown in Figure 7c, keywords with strong influence are highlighted. Each horizontal line represents a cluster, node size reflects keyword frequency, links indicate co-citation relationships, and node color corresponds to different publication years. In the early stage, research mainly focused on breakthroughs in foundational technologies and vector construction, with basic gene-editing tool studies represented by CRISPR-Cas system–related themes (e.g., #6 protospacer adjacent motif) emerging first and enabling subsequent precise bacterial engineering. Thereafter, synthetic biology (#1) as a core methodological framework became deeply integrated with research themes on genetically engineered bacteria (#2) and genetically engineered microbes (#3), forming a stable and highly productive core research trajectory. Over the past 5 years, the research frontier has clearly shifted toward translational applications and in-depth mechanistic investigations. The prominence of themes such as bacterial derivatives (#0) and drug delivery (#4) indicates a strategic transition from “engineering bacteria themselves” to “developing efficient delivery systems.” Meanwhile, gut microbiota (#5) has remained an active topic, reflecting the continuous deepening of research into the microenvironment and immune mechanisms of action (Juarez et al., 2022).

The intensity of keyword bursts also reflects the growth trends of research frontiers, hotspots, and emerging directions. As shown in Figure 7d, the strongest citation burst occurred for “priority journal” between 2017 and 2020. As an indicator of high-impact journals, this term signifies that research on genetically engineered bacteria for tumor therapy has gained recognition and focused attention from top-tier academic communities. Over the past 5 years, keywords such as engineered bacteria, neoplasm, CRISPR-Cas systems, and procedures have emerged as major burst terms, further confirming that all high-intensity historical hotspots converge on the active paradigm of engineered bacteria (Yang Y. et al., 2024), thereby guiding future research directions.

### Analysis of co-cited references

**Figure 8:**
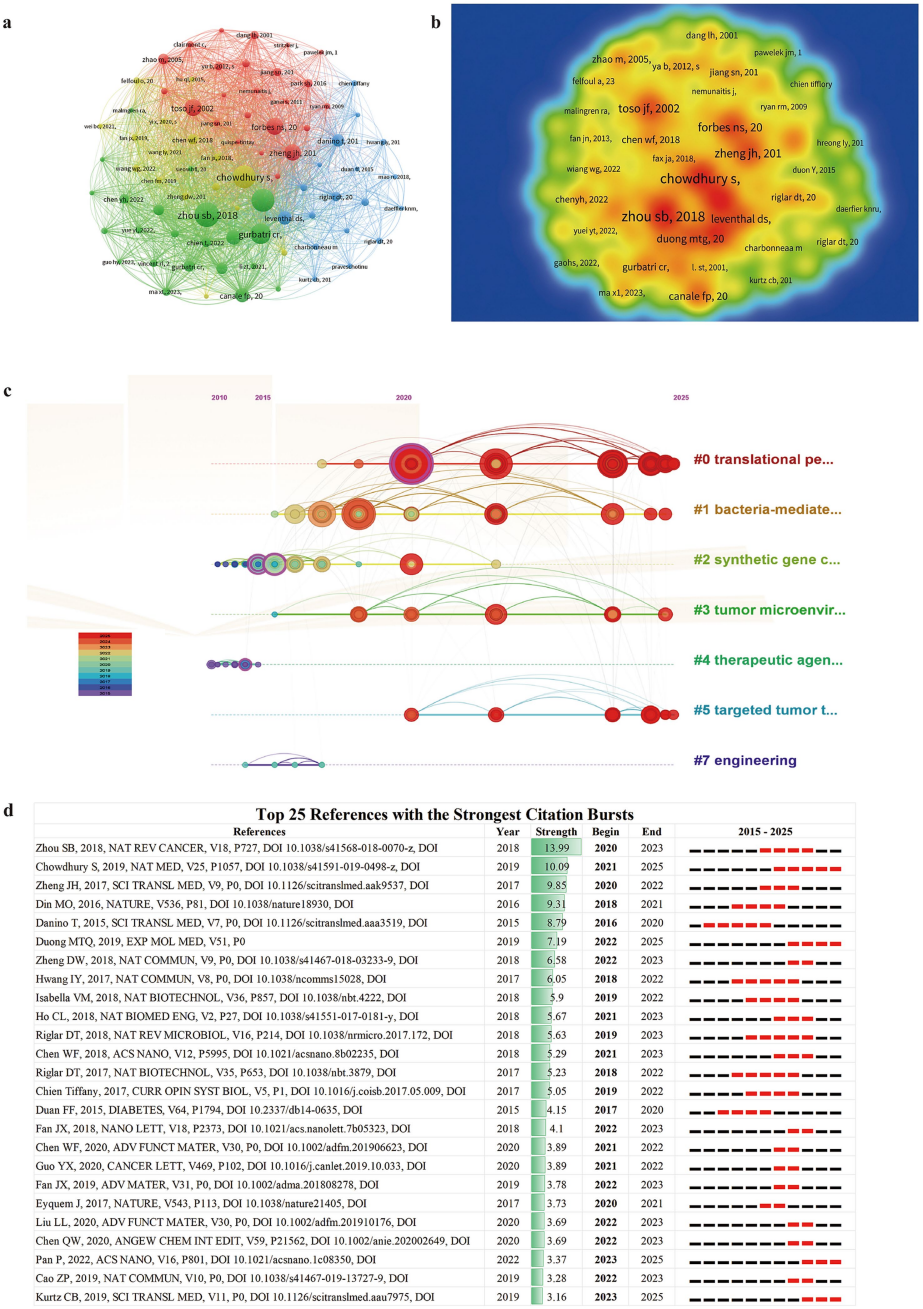
Reference analysis in genetically engineered bacteria for tumor therapy. **(a)** Co-cited reference network. **(b)** Co-citation density map. **(c)** Timeline view of co-cited literature; each horizontal line represents a cluster and node size reflects co-citation frequency, while node and link colors indicate different years. **(d)** Top 25 references with the strongest citation bursts; black bars indicate publication time and red bars indicate burst periods.

Table 5 summarizes the five most frequently co-cited references in the field of genetically engineered bacteria and tumor research. References with more than 20 co-citations were further analyzed using VOSviewer to generate network and density visualizations (Figures 8a,b). The analysis indicates that the review article entitled *“Tumour-targeting bacteria engineered to fight cancer”*, published in *Nature Reviews Cancer* by Professor Zhou Shibin’s team from the United States, ranks first with 99 co-citations (Zhou et al., 2018). This landmark publication systematically elucidates the mechanisms by which engineered bacteria achieve tumor-targeted therapy, providing a solid theoretical foundation for the development of innovative anticancer strategies while also advancing research on the clinical application of bacterial engineering technologies.

**Table 5:** Top 5 co-cited publications.

Rank	Co-cited references	Total citations	Year of publication	Journal	IF
1	Tumour-targeting bacteria engineered to fight cancer	99	2018	Nature reviews cancer	81
2	Synchronized cycles of bacterial lysis for in vivo delivery	93	2016	Nature	55
3	Programmable bacteria induce durable tumor regression and systemic antitumor immunity	92	2019	Nature medicine	50
4	Engineered probiotics for local tumor delivery of checkpoint blockade nanobodies	82	2020	Science translational medicine	16
5	Engineering the perfect (bacterial) cancer therapy	69	2010	Nature reviews cancer	81

Using CiteSpace, the co-cited references were further visualized along a temporal axis in Figure 8c, revealing the evolution of research hotspots over the past decade. Based on clustering results, these references were grouped into seven closely interconnected core knowledge clusters (such as bacteria-mediated immunotherapy, synthetic biology tools, and delivery systems). These clusters are not isolated; rather, they are interconnected and mutually reinforced through key publications. References exhibiting citation bursts are defined as those whose citation frequencies increase significantly and abruptly within a specific time period. We list the top 25 most frequently cited references over the past 10 years (Figure 8d). From 2015 to the present, the reference with the highest citation burst strength is the work by Zhou Shibin, followed by seminal studies published by the teams of Chowdhury et al. (2019), Zheng et al. (2017), Din et al. (2016), and Danino et al. (2015), all of which represent breakthroughs with substantial impact on the field.

## Discussion

Based on the bibliometric analysis of 403 research articles, we conducted an in-depth exploration of the application of genetically engineered bacteria in cancer therapy. This analysis covered various aspects, including the geographical distribution of research activity, collaboration trends, key journals and seminal literature, core keywords and research hotspots, as well as the challenges and future directions in this field.

### Geographical distribution of research activity and collaboration trends

In the field of engineered bacteria–mediated cancer therapy, China accounts for more than half of global publication output, indicating intense academic interest in this direction within the country. Teams from the United States and Europe maintain leadership in highly cited studies and pre-clinical validation; for example, the work of the Danino group (Gurbatri et al., 2022) and Han et al. (2024) and Liang et al. (2023) has attracted broad international attention. Although China leads in publication volume, citation-based indicators (Table 1) show that the average citation impact per article remains lower than that of several Western countries, particularly the United States. This mismatch is common in rapidly expanding fields where early work focuses on feasibility rather than mechanistic depth. Targeted remedies include: (i) incentivizing hypothesis-driven studies and multicentre validation, (ii) strengthening international co-submission and English-language dissemination, and (iii) fostering open-data platforms to boost visibility. Further analysis with VOSviewer reveals that engineered-bacteria cancer therapy research is dominated by high-impact scholars and core institutions, forming a transnational collaboration network centred on China, Europe and the United States. This collaborative model propels long-term development of the field and is likely to evolve toward a more efficient and diversified international cooperation system in the future.

### Key journals and influential publications

From a journal perspective, research outcomes on tumor-targeting genetically engineered bacteria are predominantly published in leading international journals such as Advanced Drug Delivery Reviews, Frontiers in Bioengineering and Biotechnology, and Journal of Controlled Release. These high-impact journals apply rigorous academic standards in manuscript selection and play a leading role in shaping the frontier of the discipline (Scott et al., 2024). Bibliometric analysis clearly demonstrates the presence of an inter-citation network within this field, indicating a virtuous academic cycle where research outputs continue to attract sustained scholarly attention (Fan et al., 2022).

Co-citation analysis highlights the significant academic value of the paper “*Tumour-targeting Bacteria Engineered to Fight Cancer*” by Zhou’s research group, published in *Nature Reviews Cancer*. This study established a comprehensive technical framework for bacterial tumor-targeting therapy, encompassing the design of engineered bacteria, elucidation of mechanisms of action, and pathways toward clinical translation. It provides a critical theoretical foundation and practical guidance for advancing research in the field of tumor-targeting genetically engineered bacteria (Zhou et al., 2018).

### Core keywords and research hotspots

Using CiteSpace to analyze keywords from 2015 to 2025, we examined the evolving research trends in this field. Between 2015 and 2019, studies primarily focused on the molecular mechanisms of genes and DNA (Kersten et al., 2016). Since 2020, research hotspots have gradually shifted toward tumor growth and therapeutic strategies, reflecting a deepening exploration of tumor progression mechanisms and methods of inhibition (Tzeng et al., 2020; Chen et al., 2022). The surge of research interest in “metastatic breast cancer” around 2021 marked a paradigm shift in therapeutic strategies for this field (Pan et al., 2022; Farheen et al., 2022). This transformation was primarily driven by breakthrough advances in antibody–drug conjugates (ADCs), exemplified by trastuzumab deruxtecan (T-DXd), which provided unprecedented survival benefits for patients with HER2-positive breast cancer (Bardia et al., 2019). In parallel, the continued refinement of precision medicine strategies has effectively overcome previous challenges of drug resistance, substantially extending patient survival. The successful expansion of the therapeutic landscape to refractory metastatic triple-negative breast cancer (mTNBC) has further broadened the beneficiary population. Therefore, the emergence of this keyword is not coincidental but rather reflects the concentrated manifestation of precision medicine’s comprehensive breakthroughs in advanced breast cancer, heralding a new era of personalized therapy and durable survival beyond traditional chemotherapy. Co-occurrence analysis of keywords further reveals the core research directions and hotspots in the field of tumor-targeting genetically engineered bacteria, which mainly encompass technology development, mechanistic investigation, and clinical application.

These bibliometric hotspots correspond closely to several key biological and translational developments in engineered bacterial cancer therapy. A range of bacterial vectors, including *E. coli*, *S. typhimurium*, and probiotic strains such as *Bifidobacterium*, have been extensively investigated because of their intrinsic ability to preferentially colonize tumor tissues and sense the hypoxic and immunosuppressive tumor microenvironment (Pan et al., 2021; Nie et al., 2024; Guo et al., 2024). Through advances in synthetic biology and gene-editing technologies, these bacteria can be programmed to deliver therapeutic molecules, immune modulators, or diagnostic signals directly within tumors, thereby enhancing therapeutic precision and reducing systemic toxicity. In recent years, several engineered bacterial systems have progressed from fundamental mechanistic studies toward translational development and early clinical exploration, highlighting their growing potential as innovative platforms for targeted cancer therapy.

### Synthetic biology and gene editing technologies

The frequent occurrence of the keywords “synthetic biology,” “engineered bacteria,” and “bacterial therapy” indirectly indicates the growing role of synthetic biology in bacterial engineering. Studies have shown that researchers have developed inducible synthetic gene circuits capable of regulating encapsulated *E. coli* Nissle 1917 (Harimoto et al., 2022). These engineered bacteria can temporarily evade immune clearance, while subsequent loss of encapsulation promotes their effective elimination from the body. This dynamic delivery strategy increased the maximum tolerated dose of bacteria by tenfold and enhanced antitumor efficacy in mouse cancer models (Wu et al., 2024; Chowdhury et al., 2019; Zhou et al., 2019). *In situ* encapsulation also improved microbial translocation within tumors and contributed to greater efficacy and safety in cancer therapy (Harimoto et al., 2022; Whitfield, 2006; Zhu et al., 2021).

In the keyword clustering analysis, terms such as “CRISPR” and “gene editing” are closely associated with genetic editing mechanisms, underscoring the indispensable role of gene editing technologies in optimizing the functions of engineered bacteria. For instance, these technologies can enhance the expression of antitumor genes or improve treatment safety (Kaczanowska et al., 2021; Mantesso et al., 2020; Chen et al., 2023). Synthetic biology and gene editing serve as foundational tools in the study of genetically engineered bacteria (Lei et al., 2024; Zhang et al., 2020; Xu and Qi, 2019). By leveraging these techniques, researchers can precisely modify bacterial genomes and rapidly construct cancer models with genotypic and phenotypic fidelity, thereby equipping bacteria with specific antitumor functionalities (Johnston and Wargo, 2024). Studies using CRISPR-Cas9 and Cre recombinase have successfully induced primary sarcomas that exhibit similar histology, growth dynamics, copy number variations, and mutational burdens, demonstrating that CRISPR-Cas9 enables the rapid generation of genetically and phenotypically consistent cancer models (Huang et al., 2017; Roper et al., 2018). In 2023, Zheng’s team published a study in *Nature Protocols* describing a precise gene insertion platform based on phage homologous recombination and the Cas3 system. This platform allows for efficient editing of key functional elements in *Pseudomonas* strains, offering critical technical support for constructing complex functional modules in engineered bacteria (Zheng et al., 2023). These gene editing strategies open promising avenues for the development of next-generation bacterial therapies.

### Bacterial therapy and tumor targeting

Bacterial therapy has emerged as a novel antitumor strategy owing to the intrinsic tumor-targeting properties of certain bacteria (Pan et al., 2021; Nie et al., 2024; Guo et al., 2024). *E. coli* and *S. typhimurium* are commonly used vectors due to their ability to selectively target tumor tissues and their relevance in vaccine development (Yang S. et al., 2024). The co-occurrence of keywords such as “*E. coli*,” “*S. typhimurium*,” “tumor targeting,” and “vaccine” further supports their potential applications in tumor-specific therapy and cancer vaccine design. These bacteria can sense the hypoxic, acidic, and immunosuppressive microenvironment of tumors, and through genetic engineering, can be programmed to precisely regulate drug expression and activate therapeutic functions (Wang et al., 2024). In 2024, Yang’s team published a study in *Nature Biotechnology* reporting an engineered probiotic system capable of tumor-specific expression of an IL-18 mutant. This system induced CD8^+^ T cell- and natural killer cell-dependent immune responses and effectively inhibited tumor progression, highlighting not only the therapeutic advantages of engineered bacteria in tumor targeting but also their synergistic potential with cellular immunotherapy (Yang S. et al., 2024).

### Synergistic effects of immunotherapy and bacteria

The combination of immunotherapy and genetically engineered bacteria is a growing research focus (Leventhal et al., 2020; Cannac et al., 2022; Johnson and June, 2016; Abujarour et al., 2016). Engineered bacteria can express antigens or immune-stimulatory molecules to activate the immune system and enhance tumor targeting (Luo et al., 2024; Lamontagne et al., 2023; Liang et al., 2021). For example, Vincent et al. engineered non-pathogenic *E. coli* to colonize tumors and deliver synthetic antigens, effectively “marking” tumors for recognition. CAR T cells were then designed to target these markers, enabling precise tumor eradication in breast and colorectal cancer models upon bacterial administration (Vincent et al., 2023).

### Drug delivery and nanotechnology

Optimizing drug delivery systems can significantly enhance the therapeutic efficacy of genetically engineered bacteria. Recent studies have explored the integration of nanotechnology with bacterial therapy to improve delivery efficiency. Feng’s team developed a strategy involving pH-responsive, charge-reversible nanomaterials to encapsulate engineered bacteria. This approach enhances bacterial accumulation and penetration within tumor tissues while minimizing retention in normal tissues, thereby improving targeting precision and treatment safety (Feng et al., 2022). The combination of nanotechnology and bacterial therapy has also shown promise in reducing postoperative recurrence and improving overall therapeutic outcomes. Wang’s group constructed a synergistic platform composed of genetically engineered *E. coli* and multifunctional nanoparticles. The modified *E. coli* specifically migrates to hypoxic tumor regions and expresses gas vesicle genes, which facilitates ultrasound imaging and enables focused ultrasound ablation surgery (FUAS) in a combined therapeutic strategy (Allemailem, 2021).

### Photothermal therapy and multimodal treatment

Photothermal therapy (PTT) combined with genetically engineered bacteria represents a promising emerging strategy for cancer treatment. The association between the keywords “photothermal therapy” and “tumor growth” suggests that the synergistic application of engineered bacteria with novel therapeutic modalities may offer innovative approaches to suppress tumor progression. Engineered bacteria can be modified to carry photosensitizers that selectively target tumors and generate localized hyperthermia upon light irradiation, effectively killing cancer cells (Liu et al., 2021). Recent studies have demonstrated that a biomimetic exosome-based nanosystem significantly improves therapeutic outcomes in triple-negative breast cancer. This system enhances intratumoral drug distribution, enables controlled drug release, and boosts efficacy by inhibiting HSP90 expression (Hu et al., 2025). Such multimodal combination therapies overcome the limitations of conventional monotherapies and provide more effective solutions for cancer treatment.

### Challenges and future directions

Although bibliometric analysis provides valuable insights into research trends, collaboration networks, and emerging hotspots at a macro level-offering important context for understanding genetically engineered bacteria in tumor therapy-it has several inherent limitations (Torres-Salinas et al., 2024). First, indicators like publication and citation counts may not reflect research depth, innovation, or clinical relevance. Highly cited works might signal topic popularity rather than meaningful contributions or translational value. Second, outcomes depend heavily on database selection and search strategies, which may introduce potential bias. In the present study, the dataset was derived from the Web of Science Core Collection (WoSCC) and Scopus databases; although these two platforms provide extensive coverage of peer-reviewed literature and are widely used in bibliometric research, relevant publications indexed exclusively in other biomedical databases such as PubMed/MEDLINE, Embase, or Dimensions may not have been captured. Citation practices may also be influenced by author prominence, institutional prestige, or journal impact. More importantly, bibliometric tools are descriptive and retrospective, incapable of evaluating mechanistic insights, biological feasibility, or clinical challenges. Thus, bibliometric findings should not be seen as direct evidence for assessing therapeutic efficacy or identifying scientific bottlenecks, but rather as auxiliary tools for mapping research structures, recognizing knowledge clusters, and generating new hypotheses.

On this basis, it is necessary to shift the focus of this paper from the macro-level knowledge mapping toward the micro-level biological and clinical realities, systematically reviewing the key challenges and developmental bottlenecks of genetically engineered bacteria in tumor therapy. Currently, the clinical translation of engineered bacteria in oncology faces multiple obstacles. The first challenge concerns biosafety. Although suicide switches and metabolism-dependent systems have been developed to enhance controllability, their long-term stability within the complex human immune environment remains to be validated (Wu et al., 2022; Liang et al., 2019; Gurbatri et al., 2020). Another major issue is insufficient targeting specificity; although certain bacterial strains exhibit natural advantages for tumor colonization, the heterogeneity of the tumor microenvironment affects colonization consistency and therapeutic outcomes (Savage et al., 2023). In addition, limited delivery efficiency and uneven *in vivo* distribution constrain therapeutic efficacy. Previous studies have demonstrated that engineered bacteria exhibit pronounced tissue tropism and time-dependent biodistribution. For instance, Savage et al. (2023) in *Science Advances*, tracked the spatiotemporal distribution of chemokine-expressing engineered bacteria in mice and observed high-density colonization within the tumor core region within 24 h, alongside remodeling of the tumor immune microenvironment through chemokine secretion to enhance local immune cell recruitment (Savage et al., 2023). Although current strategies, such as the use of nanomaterials or cellular carriers, can improve delivery efficiency, they also introduce additional complexity and costs (Feng et al., 2022). Critically, no engineered bacterial therapy has yet been approved for cancer treatment, and regulatory agencies remain cautious concerning environmental safety, genetic stability, and potential long-term adverse effects. Furthermore, the intricate interactions between engineered bacteria, the host immune system, and the microbiota remain incompletely understood, with individualized immune responses and microbial interference potentially impacting application safety.

Therefore, future research should not only deepen mechanistic investigations but also strengthen interdisciplinary collaboration, focusing on several critical directions: *in vivo* biosafety validation, optimization of scalable manufacturing processes, comprehensive pharmacokinetic evaluations, analysis of individual variations in immune responses, and the establishment of standardized clinical trial pathways, thereby accelerating the translation of engineered bacteria from laboratory research to clinical application.

## Data Availability

Publicly available datasets were analyzed in this study. This data can be found here: the analytical data for this study were sourced from the Web of Science and Scopus databases. Due to the data use terms of the publishers, the original data files cannot be publicly shared. The complete search strategy has been provided in the manuscript to ensure transparency and reproducibility of the research. Requests to access the anonymised participant data should be directed to the corresponding author.
