# Supplementary material for: Bibliometric analysis of genetically engineered bacteria in tumor therapy
Source: Front Microbiol. 2026 Apr 21;17:1794032. doi: 10.3389/fmicb.2026.1794032 (PMC13141310; doi:10.3389/fmicb.2026.1794032)
Supplement: Supplementary file 1 [file Table_1.docx]

**Supplementary Table S1-Complete search strategy**

| **Database** | **Search Query** |
| --- | --- |
| WoSCC | TS= (("genetically engineered bacteria" OR "genetic engineering bacteria" OR "engineered bacteria") AND ("cancer therapy" OR "tumor therapy" OR "oncology treatment")) OR (("synthetic biology" OR "gene editing") AND ("bacteria" AND ("cancer" OR "tumor"))) |
| Scopus | TITLE-ABS-KEY (("genetically engineered bacteria" OR "genetic engineering bacteria" OR "engineered bacteria") AND ("cancer therapy" OR "tumor therapy" OR "oncology treatment")) OR (("synthetic biology" OR "gene editing") AND ("bacteria" AND ("cancer" OR "tumor"))) |
